# Expression and phosphorylation of the AS160_v2 splice variant supports GLUT4 activation and the Warburg effect in multiple myeloma

**DOI:** 10.1186/2049-3002-1-14

**Published:** 2013-05-29

**Authors:** Javelin C Cheng, Samuel K McBrayer, Cristian Coarfa, Sevim Dalva-Aydemir, Preethi H Gunaratne, John D Carpten, Jonathan K Keats, Steven T Rosen, Mala Shanmugam

**Affiliations:** 1 Robert H. Lurie Comprehensive Cancer Center, Feinberg School of Medicine, Northwestern University, 303 E. Superior Street, Lurie Building 3-250, Chicago, IL 606011, USA; 2 Department of Molecular and Cellular Biology, Baylor College of Medicine, Houston, TX 77303, USA; 3 Department of Biology and Biochemistry, University of Houston, Houston, TX 77204, USA; 4 Division of Hematology and Oncology, Feinberg School of Medicine, Northwestern University, Chicago, IL 606011, USA; 5 The Translational Genomics Research Institute (TGen), Phoenix, AZ 85004, USA; 6 Current address: Department of Medical Oncology, Dana-Farber Cancer Institute, Boston, MA 02215, USA

## Abstract

**Background:**

Multiple myeloma (MM) is a fatal plasma cell malignancy exhibiting enhanced glucose consumption associated with an aerobic glycolytic phenotype (i.e., the Warburg effect). We have previously demonstrated that myeloma cells exhibit constitutive plasma membrane (PM) localization of GLUT4, consistent with the dependence of MM cells on this transporter for maintenance of glucose consumption rates, proliferative capacity, and viability. The purpose of this study was to investigate the molecular basis of constitutive GLUT4 plasma membrane localization in MM cells.

**Findings:**

We have elucidated a novel mechanism through which myeloma cells achieve constitutive GLUT4 activation involving elevated expression of the Rab-GTPase activating protein AS160_v2 splice variant to promote the Warburg effect. AS160_v2-positive MM cell lines display constitutive Thr642 phosphorylation, known to be required for inactivation of AS160 Rab-GAP activity. Importantly, we show that enforced expression of AS160_v2 is required for GLUT4 PM translocation and activation in these select MM lines. Furthermore, we demonstrate that ectopic expression of a full-length, phospho-deficient AS160 mutant is sufficient to impair constitutive GLUT4 cell surface residence, which is characteristic of MM cells.

**Conclusions:**

This is the first study to tie AS160 de-regulation to increased glucose consumption rates and the Warburg effect in cancer. Future studies investigating connections between the insulin/IGF-1/AS160_v2/GLUT4 axis and FDG-PET positivity in myeloma patients are warranted and could provide rationale for therapeutically targeting this pathway in MM patients with advanced disease.

## Findings

The Rab-GTPase activating protein (Rab-GAP) AKT substrate of 160 kDa (AS160) plays a critical role regulating the tethering and fusion of GLUT4-containing vesicles with the plasma membrane [1,2]. AS160 is the product of the gene *TBC1D4*, which belongs to the *TBC1* (Tre-2, BUB2p, and Cdc16p) gene family of Rab-GAPs. AS160 contains numerous phosphorylation sites, including the critical residues Ser588 and Thr642 [1]. In the presence of activating stimuli, phosphorylation of AS160 by the AGC kinases such as RSK1, SGK1, or PKB/AKT [3], leads to inactivation of the Rab-GAP domain to allow GTP-loaded Rab proteins to ferry GSVs to the PM. AS160 also functions as a positive regulator of GLUT4 trafficking [4]. Specific N terminal domains in AS160 facilitate interaction of AS160 with PM phospholipids, bringing GLUT4-GSVs in proximity to the PM. The proximity of AS160 to active kinases such as AKT in the PM promotes phosphorylation and inactivation of AS160-Rab GAP activity facilitating insertion of these proximal GLUT4-GSVs into the PM [4].

Interestingly, a novel AS160 splice variant was recently discovered (termed AS160_v2 or AS160 variant 2) which lacks exons 11 and 12 and exhibits a broad expression profile in human tissues [5]. The lack of these coding sequences appears to imbue the AS160_v2 protein with an increased permissiveness toward GLUT4 trafficking to the cell surface without the exclusion of any known phosphorylation sites or protein subdomains [5]. Importantly, it has been demonstrated that ectopic expression of AS160_v2 in rat L6 myotubes enhances GLUT4 PM localization and increases glucose consumption rates by 30% to 40% during insulin or IGF-1 treatment [5]. Given the importance of AS160 in the regulation of GLUT4 trafficking, the identification of a splice variant of AS160 displaying a diminished propensity for GLUT4 retention, and the knowledge that the AGC kinases AKT [6], RSK [7], and SGK [8] are constitutively active in myeloma, we sought to determine whether AS160 deregulation contributes substantively to the basal activation of GLUT4, that we have previously demonstrated in myeloma cells [9].

### AS160_v2 is expressed in several multiple myeloma cell lines but is absent in normal B lymphocytes

**Figure 1:**
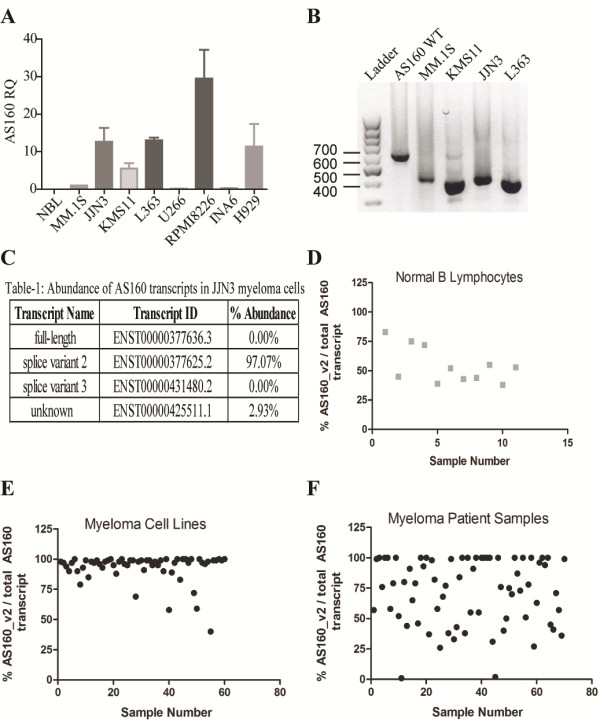
**AS160_v2 is selectively upregulated in a subset of multiple myeloma cell lines and patient samples.** (**A**) AS160 transcript levels in NBL and a panel of 8 MM cell lines assessed by qRT-PCR. Primers directed towards exon 1, common to full length and AS160_v2 transcripts were used in the quantitative RT-PCR analysis. Bars represent mean ± SD of three repeats; (**B**) Agarose gel electrophoresis of PCR products generated with primers flanking exons 11 and 12 from cDNA of MM.1S, KMS11, JJN3, and L363 cell lines. PCR product from a reaction using a vector containing full-length AS160 cDNA as template is included as a control; (**C**) Analysis of AS160 transcript abundance in the JJN3 cell line. AS160_v2 abundance in NBL (**D**), MM cell lines (**E**), and MM patient sample transcriptomes (**F**). Samples lacking AS160_v2 (12 of 82 normal B cells) were excluded.

Quantitative RT-PCR performed on a panel of multiple myeloma (MM) cell lines revealed an up-regulation of AS160 at the transcript level in JJN3, KMS11, L363, RPMI8226, and H929 cells (Figure 1A), while MM.1S, U266, and INA6 exhibit low levels of AS160 mRNA expression, comparable to normal B lymphocytes (NBL). The identity of the AS160_v2 isoform was verified by PCR analysis. A region of the AS160 transcript spanning exons 11 and 12 was amplified from cDNA generated from MM cell lines and full-length AS160 cDNA, as a control. The predominant PCR product amplified from the MM cell line cDNA was significantly shorter than the product from the vector; consistent with the 189 bp length of exons 11 and 12 (Figure 1B). Sequencing of the PCR products confirmed the absence of exons 11 and 12 [5]. Furthermore, RNA-Seq transcriptomic analysis of the JJN3 myeloma cell line confirmed complete sequence identity with the AS160_v2 reference sequence [5]. In addition to the full length AS160 sequence and AS160_v2 sequences, six additional AS160 transcripts have been identified and deposited in the NCBI database. We quantified the reads associated with the AS160 full-length, AS160_v2 splice isoform and additional transcripts and found that AS160_v2 constitutes 94.8% of all transcripts associated with this gene in the JJN3 cell line (Figure 1C). Moreover, analysis of 60 other MM cell line transcriptomes demonstrated that AS160_v2 accounted for greater than 95% of all AS160 expression (Figure 1E) in the vast majority of the tested cell lines.

Importantly, in an analysis of MM patient sample transcriptomes we found that 38 of 82 (46%) of the samples demonstrated greater than 75% of AS160 transcripts to be AS160_v2 (Figure 1F). Analysis of 11 normal B cell transcriptomes revealed significantly lower AS160_v2 expression (~38% to 55% of total transcript abundance, Figure 1D). A t-Test comparing myeloma patient samples and NBL samples for AS160_v2 abundance demonstrated that the expression of AS160_v2 is significantly higher in the MM cells (ρ = 2.5×10^-43^). NBLs and MM patient samples and cell lines did not express full-length AS160 transcript (from analysis of the RNA-Seq data sets – data not shown) normally expressed in muscle and adipose tissue.

### AS160_v2 is phosphorylated in MM cell lines and GLUT4 is removed from the PM by expression of a non-phosphorylatable, full length AS160 protein

**Figure 2:**
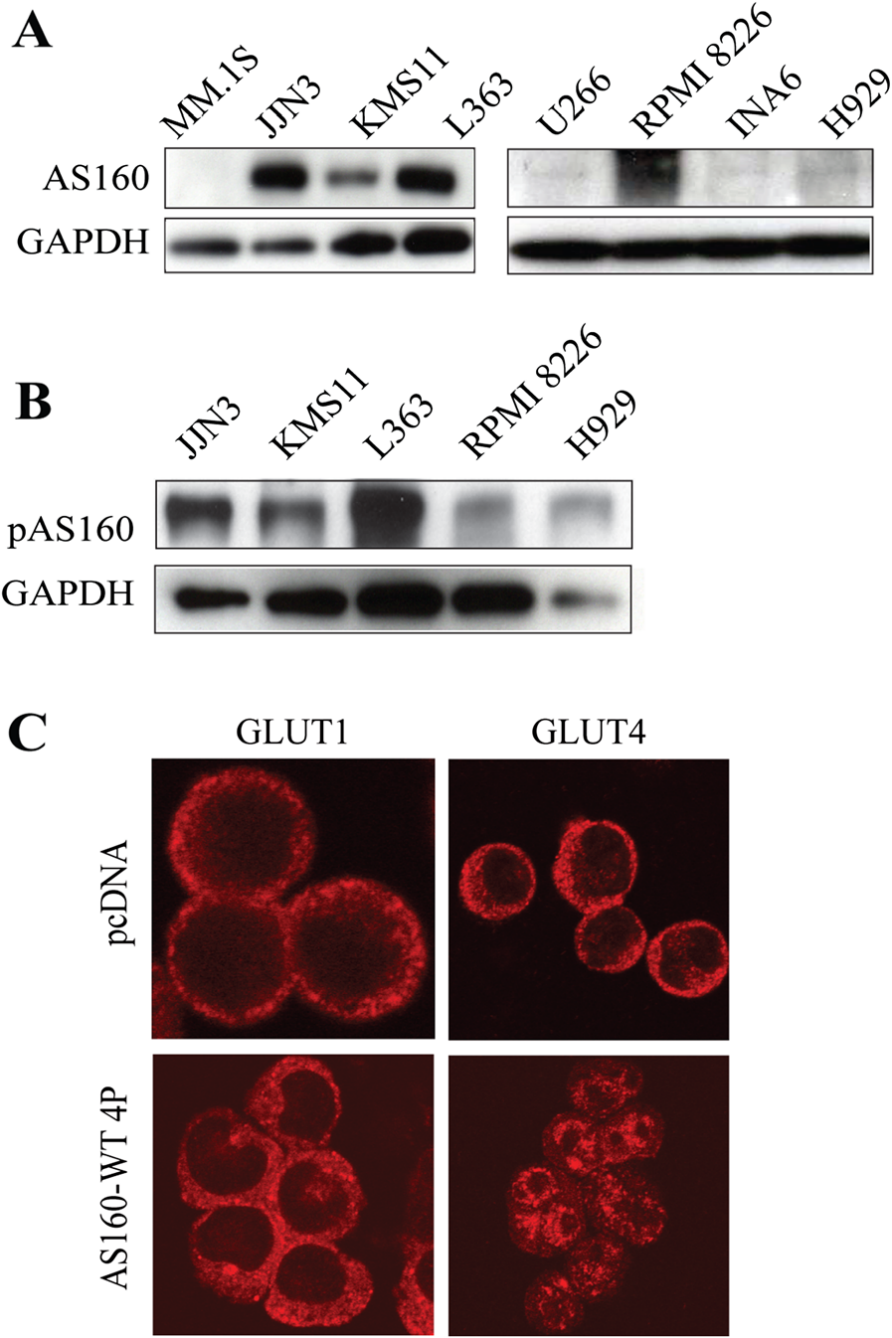
**AS160_v2 is phosphorylated in myeloma cells and introduction of a phospho-deficient AS160 mutant impairs constitutive GLUT4 plasma membrane localization.** (**A**) Immunoblot analysis of AS160 protein levels in MM cell lines; (**B**) Immunoblot analysis of pThr642 AS160 protein levels in MM cell lines; (**C**) GLUT1 and GLUT4 localization in MM.1S cell line transiently co-transfected with GFP and either empty vector (pcDNA) or full-length AS160-4P; 48 h after transfection, GFP-positive cells were sorted and imaged for GLUT1 and GLUT4 via confocal immunofluorescence microscopy. Images and immunoblots are representative of three individual repeats.

Immunoblot analysis revealed that AS160_v2 is constitutively phosphorylated on Thr642 in JJN3, KMS11, L363, and RPMI8226 cells (Figure 2B) under basal conditions. Thr642 is a residue known to be phosphorylated by AKT [3] during insulin stimulation in myocytes and adipocytes, and necessary for maintenance of GLUT4 activity and whole-body glucose homeostasis in mice [10].

We then examined the effects of expressing full-length AS160 and impairing AS160 phosphorylation on GLUT4 trafficking by testing the well-characterized AS160-4P mutant [1]. To avoid the potentially confounding effects of endogenous AS160_v2 protein, we selected MM.1S cells for these studies based on their low baseline AS160 expression. MM.1S cells were transiently transfected with the AS160-4P construct, which contains alanine substitutions at four critical phosphorylation sites required for GLUT4 trafficking to the surface or an empty vector [1]. Cells were then stained for GLUT1 and GLUT4 and visualized via confocal immunofluorescence microscopy to evaluate subcellular localization profiles. Since GLUT1 is constitutively localized on the PM [9], it serves as a marker for PM bound proteins as well as a negative control, as it remains unchanged upon introduction of AS160-4P (Figure 2C). In contrast, AS160-4P-expressing MM.1S cells revealed an intracellular sequestration of GLUT4, based on discrete, punctate cytosolic staining which contrasts with that observed in cells transfected with control pcDNA (Figure 2C). These results demonstrate that ectopic expression of a full-length, phospho-deficient AS160 mutant is sufficient to impair constitutive GLUT4 cell surface residence, which is characteristic of MM cells [9].

### Suppression of AS160_v2 expression decreases glucose consumption and proliferation by reducing GLUT4 PM content

**Figure 3:**
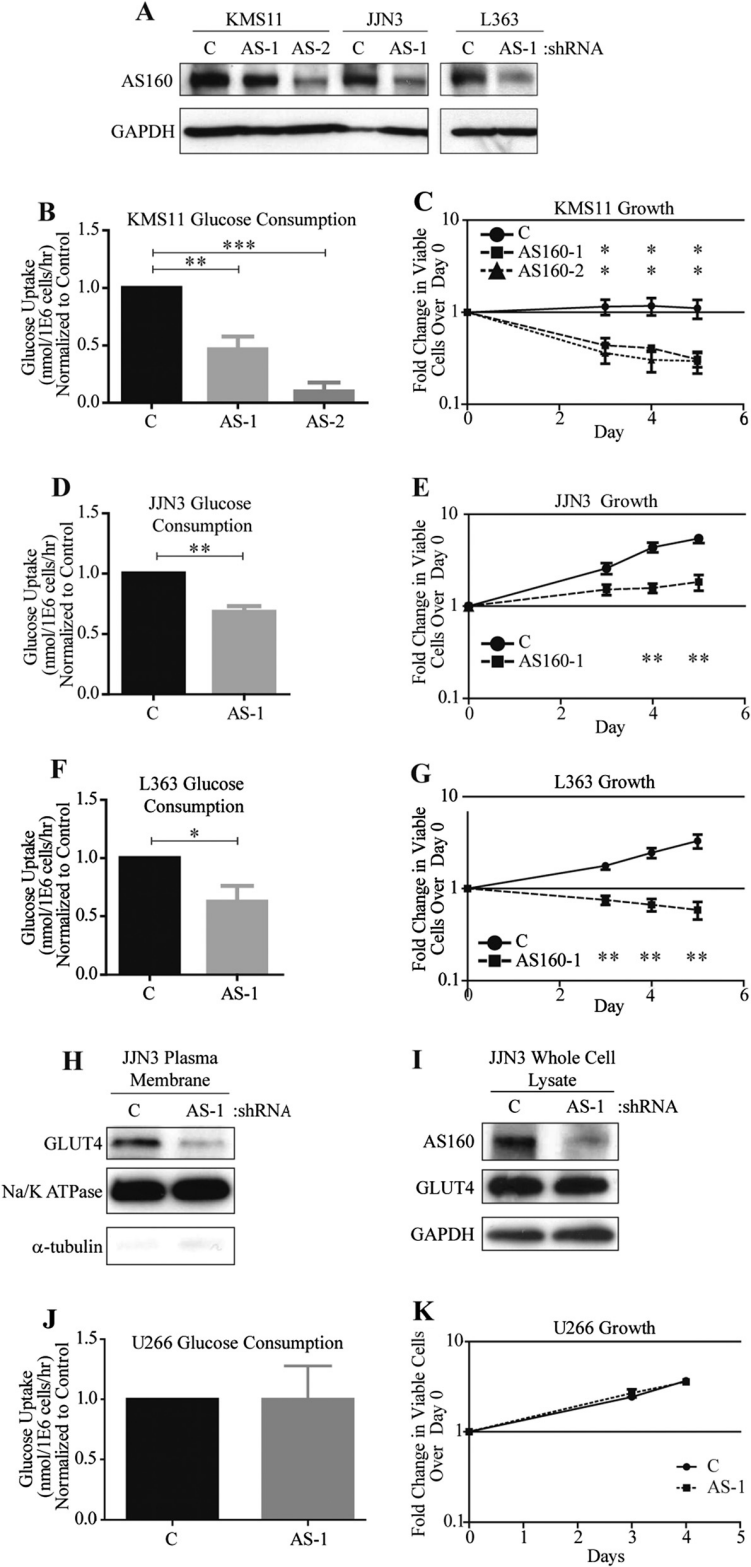
**AS160 knockdown suppresses glucose consumption and inhibits cell growth by reducing PM GLUT4 content in KMS11, JJN3, and L363 but not in U266 cells.** (**A**) Immunoblot analysis verifying AS160 knockdown with two distinct AS160 targeting shRNA’s. Image representative of three individual repeats is presented; (**B**, **D** and **F**) Glucose uptake rates in KMS11, JJN3, and L363 cells, respectively, containing non-target control, AS-1 and AS-2 shRNAs. Rates were determined at 0 and 5 h after incubation in 5 mM glucose; (**C**, **E** and **G**) Growth rates in KMS11, JJN3, and L363 cell lines containing non-target control, AS-1 and AS-2 shRNAs, show trypan blue excluded viable cell counts at 0, 3, 4, and 5 days; (**H**) Immunoblot analysis of plasma membrane proteins from JJN3 cells infected with non-target control and AS-1 shRNAs for GLUT4 and Na/K and α-tubulin loading controls; (**I**) Immunoblot analysis for AS160, GLUT4, and GAPDH loading control from whole cell lysates in JJN3 cells treated with non-target control and AS-1 shRNAs. Immunoblots are representative of two individual repeats; (**J**) Glucose uptake rates in U266 containing non-target control and AS-1 shRNA; (K) Growth rates in U266 containing non-target control and AS-1 shRNAs, show trypan blue excluded viable cell counts at 0, 3, and 4 days. Bars and points represent mean ± SD of three repeats; *P <0.05, **P <0.01, ***P <0.001.

Cell lines that basally up-regulate AS160_v2 (KMS11, JJN3, and L363) were stably transduced with pLKO.1 lentiviral vectors expressing shRNAs specific to AS160 (AS-1, AS-2) or noncoding control (C). The loss of AS160_v2 protein (Figure 3A) universally reduces glucose consumption three days post-infection (Figure 3B, D and F). Inhibition of glucose transport by AS160_v2 silencing is associated with cytotoxic effects in KMS11 and L363 cell lines and reduces cell growth in JJN3 cells (Figure 3C, E and G). Expression of an alternative AS160 shRNA (labeled AS-2) also reduces glucose uptake and induces cell death (Figure 3B-C). PM proteins were extracted from JJN3 cells expressing C and AS-1 shRNAs to correlate GLUT4 PM localization with the expression of AS160-V2. Immunoblot analysis demonstrated reduced GLUT4 content on the cell surface in cells expressing the AS-1 shRNA, while whole-cell GLUT4 levels remained constant (Figure 3H-I). The outcomes of RNAi experiments performed in the KMS11, JJN3, and L363 cell lines show that AS160_v2 silencing closely recapitulates the effects of GLUT4 suppression and glucose deprivation, with KMS11 and L363 cells undergoing substantial cell death in response to these perturbations while JJN3 cells consistently display a growth defect with maintained viability [9]. As an additional negative control, we transduced U266 cells with C and AS-1 shRNAs. Since U266 cells express very low levels of AS160_v2, there is no impact on glucose uptake or cell growth (Figure 3J-K).

## Conclusions

In sum, we have elucidated a novel mechanism through which MM cells achieve constitutive GLUT4 activation to promote the Warburg effect. Our findings corroborate those described by Baus and colleagues regarding the stimulatory function displayed by phosphorylated AS160_v2 towards GLUT4 trafficking [5]. In their study, the authors demonstrate that ectopic expression of human AS160_v2 cDNA in AS160-negative L6 rat myoblast cells potentiates GLUT4 cell surface localization and cellular 2-deoxy-D-glucose uptake in a signaling-dependent manner. It has also been shown that GLUT4 translocation is suppressed in insulin-treated 3T3-L1 adipocytes following RNAi-mediated silencing of full-length AS160 [11,12]. In line with these *in vitro* findings, a loss-of-function nonsense mutation in the *TBC1D4* gene in a severely insulin-resistant *Acanthosis nigricans* patient was reported [13]. Interestingly, expression of the resulting truncated AS160 protein (R363X) in 3T3-L1 adipocytes recapitulated the aberrations associated with AS160 silencing, thus connecting impaired AS160 function with diminished GLUT4 activity under insulin-replete conditions.

More recently, studies in adipocytes and soleus isolated from AS160^−/−^ mice demonstrated impaired insulin stimulated glucose uptake and reduced insulin-stimulated GLUT4 PM translocation [14]. Interestingly, the AS160^−/−^ adipocytes and soleus also exhibited reduced GLUT4 content suggesting a potential role for AS160 in regulating insulin-sensitivity by modulation of GLUT4 content [14]. These studies strongly support a model of AS160 function in which both full-length AS160 and AS160_v2, when phosphorylated, augment the actions of insulin to promote GLUT4 cell surface localization. We hypothesize, however, that under identical, stimulatory signaling conditions (e.g., insulin receptor activation), AS160_v2 more robustly promotes GLUT4 trafficking in comparison to full-length AS160. This hypothesis is based on the findings that the fractional increase observed in insulin-stimulated 2-DG uptake by L6 myotubes overexpressing AS160_v2 [5] is greater than the fractional decline in insulin stimulated 2-DG transport by 3T3-L1 adipocytes displaying knockdown of full-length AS160 [12]. We acknowledge, however, that further studies comparing these two splice variants under identical experimental conditions are necessary to extract meaningful information regarding their distinct functionalities. Furthermore, interrogation of the functional consequences of inclusion of exons 11 and 12 in the AS160 protein is paramount to understanding the differences between isoforms.

While the biological functions of the additional AS160 truncated transcripts detected in NBL and MM patient samples are unknown, one can speculate that possible maintenance of Rab-GAP activity in these transcripts may increase retention of GLUT4 or regulate the propensity of GLUT4 PM trafficking in response to stimulatory signals. Since insulin and IGF-1 are important myeloma growth factors [15,16] there may exist a selective pressure in MM to upregulate genes that couple insulin/IGF-1 signaling to metabolic effectors that promote aerobic glycolysis.

We have previously demonstrated that some MM cell lines, like MM.1S and U266, exhibit constitutive GLUT4 cell surface localization even in the absence of AS160 expression [9], supporting investigation of alternative, AS160-independent mechanisms of GLUT4 activation in MM. One remaining unresolved issue pertains to the identity of the kinases responsible for AS160 phosphorylation in the context of myeloma. Given a recent report detailing the widespread detection of tumor-specific AS160 phosphorylation in breast cancer biopsies [17], investigation of the AS160/GLUT4 axis and further identification of the upstream activating kinases in tumor cells will serve to clarify the role of AS160 deregulation in supporting the Warburg effect and the malignant phenotype.

## Experimental procedures

### Cell culture

MM.1S (generated in our laboratory), KMS11, JJN3, L363, H929, and INA6 (provided by Dr. M. Kuehl (NCI, Bethesda, MD), and RPMI8226 and U266 cell lines American Type Culture Collection (ATCC), VA, U.S.A were cultured in RPMI 1640 (Invitrogen) supplemented with 10% heat-inactivated FBS, 2 mM L-glutamine, 100 U/mL pen/strep, and 2.5 μg/mL Amphotericin B at 37°C in 5% CO_2_.

### B cell isolation

Normal B lymphocytes were purified from peripheral blood mononuclear cells using the EasySep™ Negative Selection Human B Cell Enrichment Kit (STEMCell Technologies, BC, Canada) as previously described [9].

### RNA extraction

RNA was extracted from MM cell lines and normal B lymphocytes using RNeasy™ Mini Kit (Qiagen) per manufacturer’s instructions.

### Reverse transcription of RNA extracts and relative quantitative real time PCR

Total RNA isolated from cell lines and normal B lymphocytes was transcribed into cDNA using Multiscribe™ Reverse Transcriptase (Applied Biosystems) according to the manufacturer’s instructions. Quantitative RT-PCR experiments were performed on an ABI 7900HT Fast Real-Time PCR system (ABI) using the Taqman Universal Master Mix (ABI). YWHAZ, RPL13A, and EIF4A primer/probe sets (Primerdesign Ltd, U.K) were used as endogenous controls across MM cell lines. Primer/probe sets specific for Exon 1 and Exons 5–7 of AS160 (ABI) were used for relative quantification of AS160 transcript levels across MM cell lines.

### Sequencing the AS160.V2 junction

The following primers were used for PCR amplification and sequencing of the AS160.V2 junction and span a region 137 bp upstream and 219 bp downstream of Exon 11 and 12 AS160_v2 deletion: FWD – AS160_Junction, 5′ – AACGTTTCCCGAAGAGGATTCCGA – 3′ and REV-AS160_Junction, 5′–ACAGGAATACAACCAGCGGTTCCT–3′ (Integrated DNA Technologies). cDNA from MM lines and NBLs were subjected to PCR amplification. A vector containing full-length AS160 (FL-AS160) (kindly provided by Dr. G Lienhard, Dartmouth College, NH) was used as a positive control and amplified with one round of PCR. For each round, 800 ng cDNA and 50 ng vector DNA were amplified under the following conditions: 1× Thermopol buffer (containing 20 mM Tris–HCl, 10 mM (NH_4_)_2_SO_4_, 10 mM KCl, 2 mM MgSO_4_, 0.1% Triton X-100, pH 8.8 at 25°C), 0.8 mM total dNTPs, 100 ng primers, and 2 U Vent Polymerase (New England Biolabs). DNA was subjected to PCR conditions as follows: 30 cycles of denaturing at 95°C for 30s, primer annealing at 52°C for 30s, extension at 72°C for 1 minute, and final extension at 72°C for 10 minutes. DNA fragments were analyzed by agarose gel electrophoresis, excised using a Gel Extraction Kit (Qiagen) and sequenced with same PCR primers using an ABI 3730 High-Throughput DNA Sequencer (Applied Biosystems) (Center for Genetic Medicine, Northwestern University).

### Protein extraction

Whole cell protein lysates were prepared as previously described [9]. Plasma membrane proteins were extracted from 50 × 10^6^ cells homogenized at 30 Hz for 3 minutes using TissueLyser LT (Qiagen) and plasma membrane proteins were extracted using the Plasma Membrane Protein Extraction Kit (BioVision) according to the manufacturer’s instructions.

### RNA-Seq

mRNA-Seq sequencing of the JJN3 MM cell line yielded 49 million tags. The data was mapped onto the human genome, build UCSC hg19/NCBI 37, with the suite of tools bowtie2 and tophat2 [18,19]; 40 million tags were aligned onto the reference genome (81% mapping ratio). Transcript abundance was assessed using cufflinks and the GENCODE [20] gene definition version 14.0; GENCODE includes definitions for both the AS160 and AS160_v2 variants of TBC1D4. Access to Multiple Myeloma Research Consortium patient sample and cell line RNA-Seq data sets were obtained from Dr. Jonathan Keats, TGen. NBL RNA-Seq data sets were obtained from the NCBI Gene Expression Omnibus (GEO) repository.

### SDS-PAGE and immunoblot analysis

Extracted proteins were separated by SDS-PAGE using Novex 8-16% Tris-glycine Mini gels (Invitrogen) or 4-15% Mini-Protean TGX Precast Gels (Bio-Rad) and transferred onto nitrocellulose or PVDF membranes and detected by Amersham™ ECL Plus Western Blotting Reagents (GE Healthcare), as previously described [9]. The following primary antibodies were used: α-tubulin (Santa Cruz Biotechnology Inc.), total AS160 anti-serum (Millipore), pThr 642 AS160 (Invitrogen), GAPDH (Santa Cruz Biotechnology), GLUT4 anti-serum (provided by Dr. S. Cushman, National Institute of Diabetes and Digestive and Kidney Diseases, Bethesda, MD), Na^+^/K^+^ ATPase Plasma Membrane Marker (Abcam), secondary mouse and rabbit antibodies conjugated to horseradish peroxidase (Cell Signaling Technology).

### Transient transfection into MM.1S

MM.1S cells were co-transfected with either empty pcDNA vector or a full length AS160-4P (FL AS160-4P) construct (Dr. G. Lienhard, Dartmouth college, NH) and a GFP expressing plasmid (pmax GFP™ plasmid, Amaxa) using the Amaxa Nucleofector Kit V and the Amaxa Nucleofector 2b Device, with program O23 (Lonza) per manufacturer’s instructions. After 48 h of culture GFP positive cells were sorted on a Beckman Coulter MoFlo™ XDP Cell Sorter.

### Immunofluorescence microscopy

Cells were washed in PBS and spun onto microscope slides (Shandon Cytoslide) using a Shandon Cytospin centrifuge (Thermo Fischer Scientific). Slides were fixed in 4% freshly prepared paraformaldehyde at pH 7.4, permeabilized with 0.03% saponin in PBS, and incubated with blocking buffer (10% normal goat serum containing 0.03% saponin). Cells were stained with anti-GLUT1 (Abcam) or anti-GLUT4 (Dr. S. Cushman, NIDDK, MD) and secondary antibodies in blocking buffer for 1 h at room temperature as previously described [9]. Cells were visualized at 63× (1.4 NA) oil objective with an LSM-510 Meta, Carl Zeiss confocal microscope. Image analysis was performed using the Zeiss Axio vision LE image browser.

### Lentiviral production and transduction in MM cell lines

Non-targeting CshRNA and AS160 shRNA pLKO.1 vectors (Sigma) were packaged and lentivirus generated as described previously [9]. Cells were selected in Puromycin (KMS11, 0.3 μg/mL; L363, 0.5 μg/mL; JJN3, 0.25 μg/mL; U266, 0.25 μg/mL), two days post-infection.

### Cell growth assay

Three days post-infection (day 0), an equal number of cells were plated in T25 flasks. For 0, 3, 4, and 5 day time points, cells were harvested and viable cell count was determined by Trypan Blue exclusion using Vi-Cell Viability Analyzer (Beckman-Coulter).

### Glucose consumption assay

The rate of glucose uptake was determined three days post-infection in cells incubated in 5 mM glucose RPMI 1640 medium for 5 h in a 37°C incubator with 5% CO_2_. The concentration of glucose was detected in media obtained at 0 and 5 h using the Amplex Red™ Glucose/Glucose Oxidase Assay Kit (Invitrogen) according to the manufacturer’s instructions.

## Availability of supporting data

"The data set(s) supporting the results of this article is(are) included within the article (and its additional file(s))".

## Abbreviations

MM: Multiple myeloma; NBL: Normal B lymphocytes; PM: Plasma membrane; Rab-GAP: Rab-GTPase activating protein.

## Competing interest

The authors declare no competing financial interests.

## Authors’ contributions

JCC and MS conceived and performed the research; PHG performed RNASeq on the JJN3 cell line and SDA assisted in sequence analysis; JDC and JKK provided MM patient and cell line RNA sequencing data; CC mapped, aligned, and evaluated transcript abundance in the cell lines and primary patient sample datasets. STR, SKM, JCC, SDA, and MS provided conceptual advice; SKM and MS wrote the manuscript and MS supervised the project. All authors read and approved the final manuscript.

## References

[B1] Sano H Kane S Sano E Mîinea CP Asara JM Lane WS Garner CW Lienhard GE Insulin-stimulated phosphorylation of a Rab GTPase-activating protein regulates GLUT4 translocation J Biol Chem 2003 278 17 14599 14602 10.1074/jbc.C300063200 12637568

[B2] Kane S Sano H Liu SC Asara JM Lane WS Garner CC Lienhard GE A method to identify serine kinase substrates. Akt phosphorylates a novel adipocyte protein with a Rab GTPase-activating protein (GAP) domain J Biol Chem 2002 277 25 22115 22118 10.1074/jbc.C200198200 11994271

[B3] Geraghty KM Chen S Harthill JE Ibrahim AF Toth R Morrice NA Vandermoere F Moorhead GB Hardie DG MacKintosh C Regulation of multisite phosphorylation and 14-3-3 binding of AS160 in response to IGF-1, EGF, PMA and AICAR Biochem J 2007 407 2 231 241 10.1042/BJ20070649 17617058 PMC2049023

[B4] Tan SX Ng Y Burchfield JG Ramm G Lambright DG Stöckli J James DE The Rab GTPase-activating protein TBC1D4/AS160 contains an atypical phosphotyrosine-binding domain that interacts with plasma membrane phospholipids to facilitate GLUT4 trafficking in adipocytes Mol Cell Biol 2012 32 24 4946 4959 10.1128/MCB.00761-12 23045393 PMC3510527

[B5] Baus D Heermeier K De Hoop M Metz-Weidmann C Gassenhuber J Dittrich W Welte S Tennagels N Identification of a novel AS160 splice variant that regulates GLUT4 translocation and glucose-uptake in rat muscle cells Cell Signal 2008 20 12 2237 2246 10.1016/j.cellsig.2008.08.010 18771725

[B6] Hsu J Shi Y Krajewski S Renner S Fisher M Reed JC Franke TF Lichtenstein A The AKT kinase is activated in multiple myeloma tumor cells Blood 2001 98 9 2853 2855 10.1182/blood.V98.9.2853 11675360

[B7] Anjum R Blenis J The RSK family of kinases: emerging roles in cellular signalling Nat Rev Mol Cell Biol 2008 9 10 747 758 10.1038/nrm2509 18813292

[B8] Fagerli UM Ullrich K Stühmer T Holien T Köchert K Holt RU Bruland O Chatterjee M Nogai H Lenz G Shaughnessy JD Jr Mathas S Sundan A Bargou RC Dörken B Børset M Janz M Serum/glucocorticoid-regulated kinase 1 (SGK1) is a prominent target gene of the transcriptional response to cytokines in multiple myeloma and supports the growth of myeloma cells Oncogene 2011 30 28 3198 3206 10.1038/onc.2011.79 21478911

[B9] McBrayer SK Cheng JC Singhal S Krett NL Rosen ST Shanmugam M Multiple myeloma exhibits novel dependence on GLUT4, GLUT8, and GLUT11: implications for glucose transporter-directed therapy Blood 2012 119 20 4686 4697 10.1182/blood-2011-09-377846 22452979 PMC3367873

[B10] Chen S Wasserman DH MacKintosh C Sakamoto K Mice with AS160/TBC1D4-Thr649Ala knockin mutation are glucose intolerant with reduced insulin sensitivity and altered GLUT4 trafficking Cell Metab 2011 13 1 68 79 10.1016/j.cmet.2010.12.005 21195350 PMC3081066

[B11] Brewer PD Romenskaia I Kanow MA Mastick CC Loss of AS160 Akt substrate causes Glut4 protein to accumulate in compartments that are primed for fusion in basal adipocytes J Biol Chem 2011 286 30 26287 26297 10.1074/jbc.M111.253880 21613213 PMC3143591

[B12] Eguez L Lee A Chavez JA Miinea CP Kane S Lienhard GE McGraw TE Full intracellular retention of GLUT4 requires AS160 Rab GTPase activating protein Cell Metab 2005 2 4 263 272 10.1016/j.cmet.2005.09.005 16213228

[B13] Dash S Sano H Rochford JJ Semple RK Yeo G Hyden CS Soos MA Clark J Rodin A Langenberg C Druet C Fawcett KA Tung YC Wareham NJ Barroso I Lienhard GE O'Rahilly S Savage DB A truncation mutation in TBC1D4 in a family with acanthosis nigricans and postprandial hyperinsulinemia Proc Natl Acad Sci USA 2009 106 23 9350 9355 10.1073/pnas.0900909106 19470471 PMC2695078

[B14] Lansey MN Walker NN Hargett SR Stevens JR Keller SR Deletion of Rab GAP AS160 modifies glucose uptake and GLUT4 translocation in primary skeletal muscles and adipocytes and impairs glucose homeostasis Am J Physiol Endocrinol Metab 2012 303 10 E1273 1286 10.1152/ajpendo.00316.2012 23011063 PMC3517634

[B15] Sprynski AC Hose D Caillot L Réme T Shaughnessy JD Jr Barlogie B Seckinger A Moreaux J Hundemer M Jourdan M Meissner T Jauch A Mahtouk K Kassambara A Bertsch U Rossi JF Goldschmidt H Klein B The role of IGF-1 as a major growth factor for myeloma cell lines and the prognostic relevance of the expression of its receptor Blood 2009 113 19 4614 4626 10.1182/blood-2008-07-170464 19228610 PMC2691749

[B16] Sprynski AC Hose D Kassambara A Vincent L Jourdan M Rossi JF Goldschmidt H Klein B Insulin is a potent myeloma cell growth factor through insulin/IGF-1 hybrid receptor activation Leukemia 2010 24 11 1940 1950 10.1038/leu.2010.192 20844560 PMC3141222

[B17] Jiang XH Sun JW Xu M Jiang XF Liu CF Lu Y Frequent hyperphosphorylation of AS160 in breast cancer Cancer Biol Ther 2010 10 4 362 367 10.4161/cbt.10.4.12426 20574165

[B18] Langmead B Salzberg SL Fast gapped-read alignment with Bowtie 2 Nat Methods 2012 9 4 357 359 10.1038/nmeth.1923 22388286 PMC3322381

[B19] Trapnell C Roberts A Goff L Pertea G Kim D Kelley DR Pimentel H Salzberg SL Rinn JL Pachter L Differential gene and transcript expression analysis of RNA-seq experiments with TopHat and Cufflinks Nat Protoc 2012 7 3 562 578 10.1038/nprot.2012.016 22383036 PMC3334321

[B20] Harrow J Frankish A Gonzalez JM Tapanari E Diekhans M Kokocinski F Aken BL Barrell D Zadissa A Searle S Barnes I Bignell A Boychenko V Hunt T Kay M Mukherjee G Rajan J Despacio-Reyes G Saunders G Steward C Harte R Lin M Howald C Tanzer A Derrien T Chrast J Walters N Balasubramanian S Pei B Tress M GENCODE: the reference human genome annotation for The ENCODE Project Genome Res 2012 22 9 1760 1774 10.1101/gr.135350.111 22955987 PMC3431492

